# Myosin-binding protein C N-terminal domains slow Ca^2+^ dissociation from cardiac thin filaments

**DOI:** 10.1016/j.jbc.2026.113601

**Published:** 2026-09-23

**Authors:** Garrett T. Hauck, Fiona L. Wong, Matthew M. Klass, Rhye-Samuel Kanassatega, Victoria C. Lepak, Thomas A. Bunch, Jil C. Tardiff, Brett A. Colson

**Affiliations:** 1 Department of Cellular & Molecular Medicine, University of Arizona, Tucson, Arizona, USA; 2 Department of Biomedical Engineering, University of Arizona, Tucson, Arizona, USA

**Keywords:** cardiac muscle regulation, cardiac myosin-binding protein C, actin-thin filament regulation, Ca^2+^ dissociation kinetics, troponin, phosphorylation, stopped-flow

## Abstract

The cardiac thin filament (cTF) regulates contraction through Ca^2+^ binding to, and release from, cardiac troponin C (cTnC), yet how sarcomeric accessory proteins influence these kinetics remains incompletely understood. Cardiac myosin-binding protein C (cMyBP-C) N-terminal domains interact with the thin filament and are regulated by PKA-mediated phosphorylation. To determine whether cMyBP-C alters thin filament Ca^2+^ exchange kinetics, we performed stopped-flow measurements using 2-(4´-(iodoacetamido)anilino)naphthalene-6-sulfonic acid-labeled cTnC in reconstituted cTFs. The N-terminal fragments C0-C2 and C1-C2 slowed Ca^2+^ dissociation in a concentration-dependent manner, whereas C0-C1 produced only a modest effect. PKA-mediated phosphorylation markedly attenuated the slowing by C0-C2 and C1-C2. Thus, N-terminal domain composition and phosphorylation state strongly influence cMyBP-C-mediated regulation of thin filament Ca^2+^ dissociation. In contrast, the tested cMyBP-C fragments did not alter Ca^2+^ association kinetics or Ca^2+^ dissociation from isolated troponin. Cosedimentation measurements further showed that differences in kinetic effects were not explained solely by differences in thin filament occupancy. Together, these results demonstrate that cMyBP-C N-terminal fragments selectively modulate Ca^2+^ dissociation from cTnC in myosin-free reconstituted cTFs. The absence of an effect on isolated troponin is consistent with regulation involving additional thin filament components, including actin and/or tropomyosin, although tropomyosin-dependent and -independent mechanisms remain possible. This work identifies a previously unrecognized effect of cMyBP-C N-terminal domains on cTnC Ca^2+^ dissociation in the cTF.

The cardiac thin filament (cTF) is the central Ca^2+^-regulated component of the sarcomere that controls contraction and relaxation. It is composed of actin (F-actin), tropomyosin (Tm), and the heterotrimeric troponin (cTn) complex, consisting of cardiac troponin C (cTnC), troponin I (cTnI), and troponin T. Regulation occurs *via* Ca^2+^ binding to site II of cTnC, triggering conformational changes that propagate through cTnI and troponin T to Tm. According to the three-state model, Tm transitions from the blocked (no Ca^2+^) to the closed (Ca^2+^-bound) position, relieving steric inhibition of myosin binding. Subsequent myosin interaction shifts Tm to the open position, enabling force generation (1, 2).

Cardiac myosin-binding protein C (cMyBP-C), localized to the C-zone of the sarcomere, is anchored to the thick filaments through its C-terminal domains, while its N-terminal domains extend to interact with actin and Tm. In the absence of Ca^2+^, these N-terminal regions have been shown to activate the thin filament through these interactions (3, 4). Recent cryo-EM studies revealed that the cTn core can adopt multiple conformations that depend on Ca^2+^, myosin, and the C1 domain of cMyBP-C (5). These structural ensembles, further modulated by PKA-mediated phosphorylation of M-domain, located between C1 and C2, provide phosphorylation-sensitive regulation of thin and thick filament interactions. The physiological importance of this regulation is underscored by the fact that mutations in cMyBP-C are a leading cause of genetic hypertrophic cardiomyopathy (6) and represent a target for emerging gene therapies (7).

Because cMyBP-C N-terminal domains alter thin filament regulatory structure and function, they may also influence Ca^2+^ exchange by thin filament-bound cTnC. Most previous studies have focused on cMyBP-C-dependent thin filament activation (5), whereas much less is known about whether these interactions affect Ca^2+^ dissociation, a molecular step associated with thin filament deactivation. We therefore tested whether cMyBP-C N-terminal fragments alter Ca^2+^ association and dissociation kinetics in myosin-free reconstituted cTFs using stopped-flow spectroscopy with 2-(4´-(iodoacetamido)anilino)naphthalene-6-sulfonic acid (IAANS)-labeled (at residue T53 C) cTnC. We found that C0-C2 and C1-C2 markedly slow Ca^2+^ dissociation, that phosphorylation attenuates this effect, and that C0-C1 produces comparatively modest effects. These findings identify a previously unrecognized effect of cMyBP-C N-terminal domains on thin filament Ca^2+^ dissociation kinetics.

## Results

### cMyBP-C N-terminal domains decrease the rate of Ca^2+^ dissociation from cTFs

To determine whether cMyBP-C N-terminal interactions with actin and Tm (3, 4, 8) influence Ca^2+^ handling by cTn in cTFs, we measured Ca^2+^ dissociation kinetics using stopped-flow fluorescence spectroscopy with fully reconstituted cTFs containing cTnC labeled with IAANS at residue T53 C, a previously established reporter of Ca^2+^-dependent conformational changes in the cTnC regulatory domain. In this assay, IAANS intensity decreases as Ca^2+^-dissociates from cTnC, and the rate of decrease in intensity is a measure of the dissociation rate (Fig. 1) (9, 10). Assays were performed in the presence of 3 mM total Mg^2+^, which is expected to closely approximate physiological free Mg^2+^. Ca^2+^-saturated cTFs containing 200 μM CaCl_2_ were rapidly mixed with EGTA, and fluorescence changes were monitored across increasing cMyBP-C fragment:Tm ratios (0.5:1–4:1).

**Figure 1:**
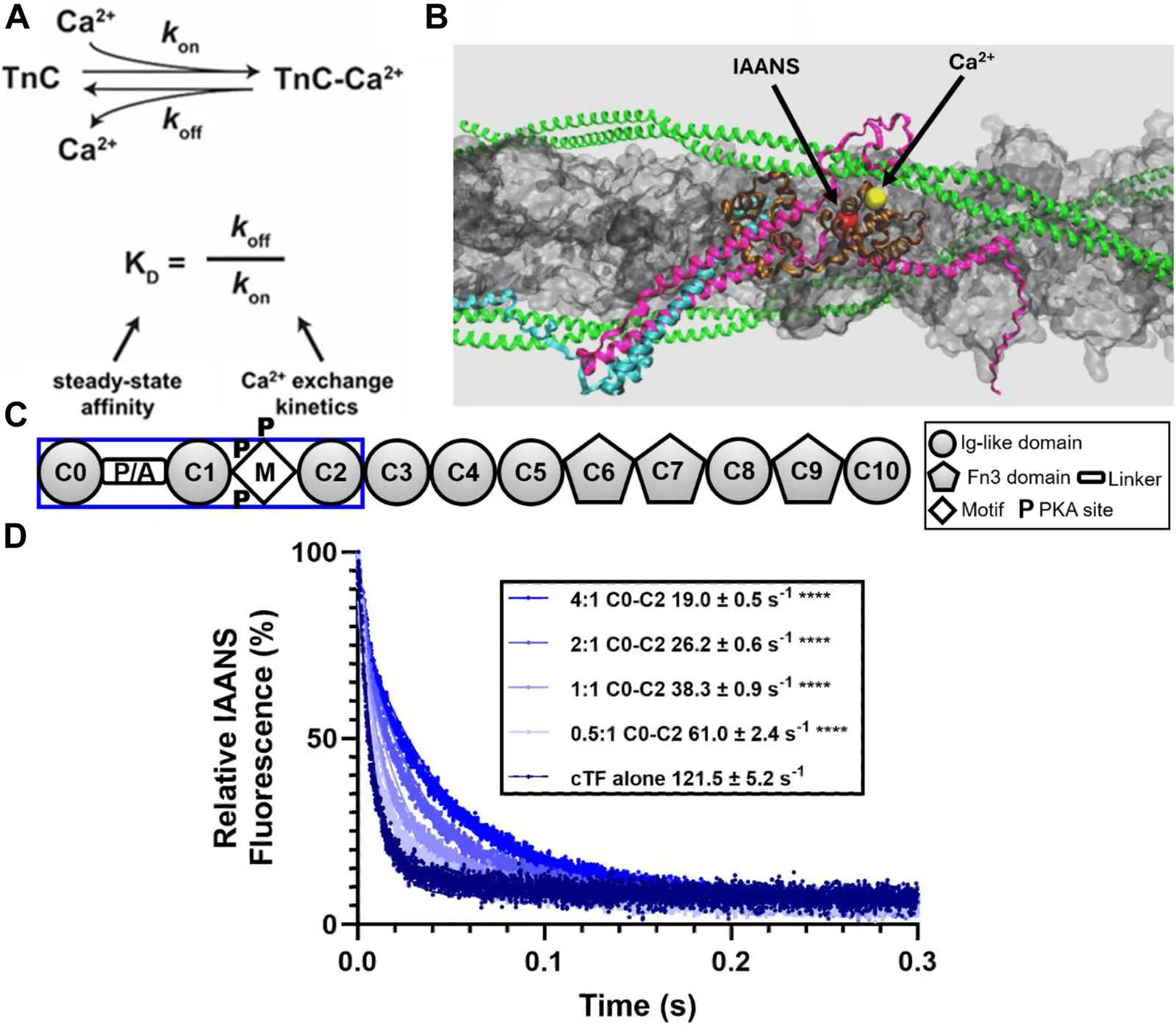
**Experimental design and effect of C0-C2 on Ca^2+^ dissociation kinetics from cTnC in cardiac thin filaments (cTFs).***A*, the relationship between the Ca^2+^ association (*k*_*on*_) and dissociation (*k*_*off*_) rate constants determines the apparent affinity (*K*_*D*_ = *k*_*off*_/*k*_*on*_). *B*, atomistic model of a single cardiac troponin complex (cTn) on a thin filament with actin (*gray*), Tm (*green*), cTnC (*brown*), cTnI (*magenta*), and cTnT (*cyan*). The IAANS probe on cTnC conjugated at T53C (*red bead*) is near the regulatory Ca^2+^-binding site (*yellow bead*). *C*, domain organization of cMyBP-C, highlighting the N-terminal fragments studied (C0-C2, C1-C2, and C0-C1) and PKA phosphorylation sites in the M-domain. *D*, representative IAANS fluorescence decays illustrating slowed Ca^2+^ dissociation in the presence of C0-C2:Tm ratios (*light periwinkle* to *royal blue traces*) compared to cTF alone (*midnight blue*) (∗∗∗∗*p* ≤ 0.0001). Statistical significance was determined by two-way ANOVA followed by Tukey’s *post hoc* test for multiple comparisons. cTnC, cardiac troponin C; cMyBP-C, cardiac myosin-binding protein C; IAANS, 2-(4´-(iodoacetamido)anilino)naphthalene-6-sulfonic acid.

Both C0-C2 and C1-C2 significantly reduced the Ca^2+^ dissociation rate compared with cTF alone (*k*_*off*_: 122 ± 5 s^−1^ for cTF; 38.31 ± 0.93 s^−1^ for 1:1 C0-C2:Tm, *p* = 5.2 × 10^−14^; 25.84 ± 0.74 s^−1^ for 1:1 C1-C2:Tm, *p* < 1.0 x 10^−15^) (Fig. 2; see also, Fig. S1 for data traces and Tables S1 and S2 for summary rates and *p*-values). This effect was markedly blunted by PKA-mediated phosphorylation of the fragments (106 ± 4 s^−1^ for 1:1 pC0-C2:Tm, *p* = 8.6 × 10^−14^; 94.35 ± 4.59 s^−1^ for 1:1 pC1-C2:Tm, *p* < 1.0 × 10^−15^). We next tested C0-C1, which lacks the M-domain and C2 region. Because C0-C1 displayed no detectable binding at the 1:1 ratio (Fig. S2), we also tested C0-C1 at 10 μM (17.5:1 C0-C1:Tm), a concentration at which binding to cTFs was detected (Fig. S2). Under these conditions, C0-C1 produced only a modest decrease in Ca^2+^ dissociation, with a *k*_off_ of 97.3 ± 2.5 s^−1^. Consistent with the absence of a PKA phosphorylation site in C0-C1, PKA treatment did not alter this effect (97.4 ± 1.8 s^−1^ for 17.5:1 pC0-C1:Tm).

**Figure 2:**
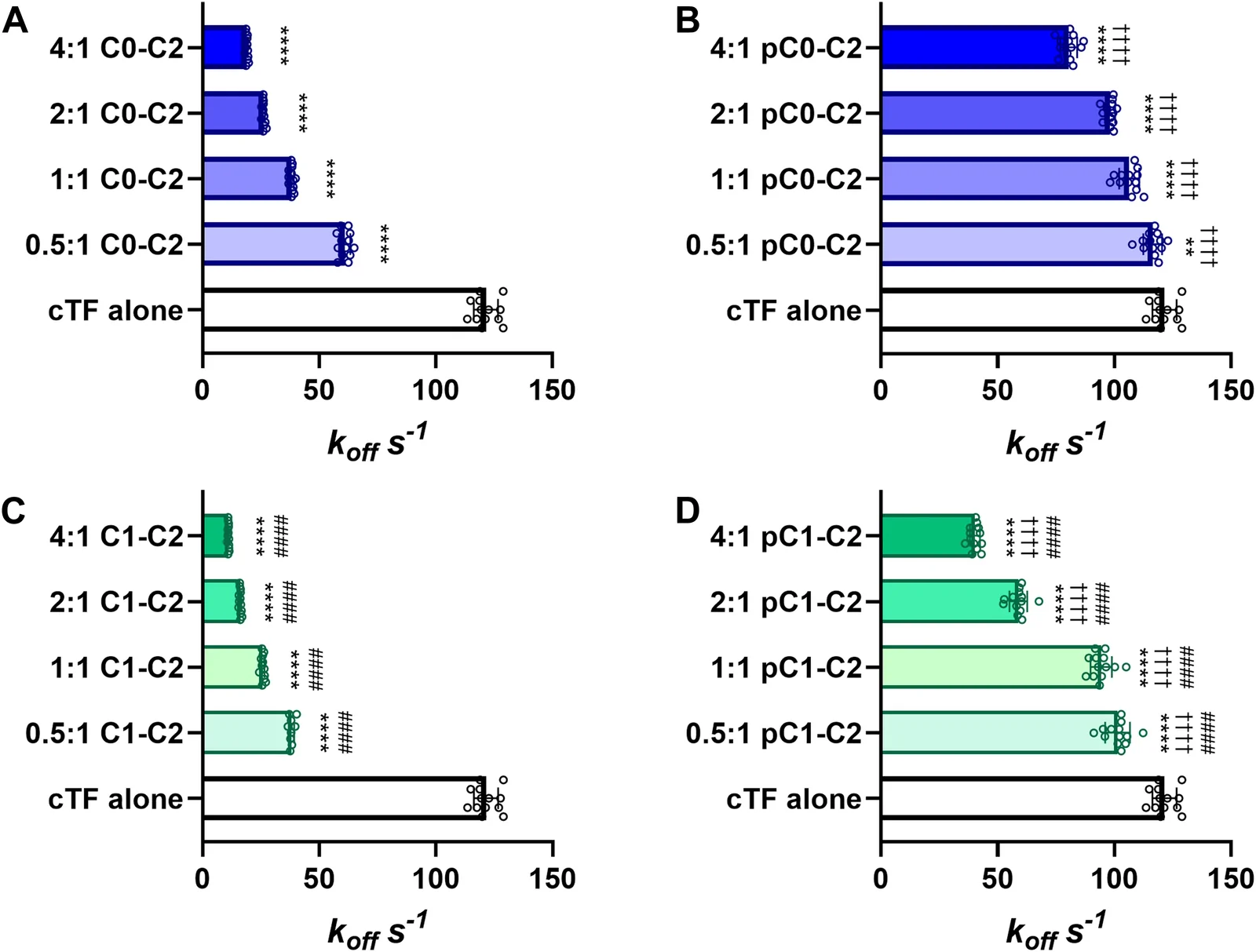
**Effects of C0-C2, C1-C2, and their phosphorylation on Ca^2+^ dissociation kinetics.** Data derived from IAANS fluorescence decays indicating Ca^2+^ dissociation from cTnC in reconstituted cTFs in the presence of unphosphorylated C0-C2 or C1-C2, or PKA-phosphorylated C0-C2 (pC0-C2) or C1-C2 (pC1-C2). *A*, dependence of dissociation rate constants (*k*_*off*_) on unphosphorylated and (*B*) phosphorylated C0-C2:Tm ratios. *C*, corresponding *k*_*off*_ values for unphosphorylated and (*D*) phosphorylated C1-C2. Data are presented as mean ± S.D. (N = 2 protein preparations, n = 13 injection averages). Statistical significance was determined by two-way ANOVA followed by Tukey’s *post hoc* test for multiple comparisons: ∗*p* ≤ 0.05 and ∗∗∗∗*p* ≤ 0.0001 for C0-C2 or C1-C2 *versus* cTF alone; ^††††^*p* ≤ 0.0001 for C0-C2 *versus* pC0-C2 or C1-C2 *versus* pC1-C2 at the same cTF ratio; ^####^*p* ≤ 0.0001 for C1-C2 *versus* C0-C2 at the same cTF ratio and PKA condition. IAANS, 2-(4´-(iodoacetamido)anilino)naphthalene-6-sulfonic acid; cTnC, cardiac troponin C; cTF, cardiac thin filament.

### cMyBP-C N-terminal domains bind cTFs

Binding of cMyBP-C N-terminal fragments to cTFs was quantified under the same buffer conditions as the kinetic measurements. C0-C2 and C1-C2 exhibited concentration-dependent binding that was reduced by PKA-mediated phosphorylation, whereas C0-C1 showed weaker binding (*p* < 1 × 10^−15^ *versus* C0-C2) (Fig. S2). Binding differs across fragments and upon phosphorylation (Fig. S2). When these binding differences are accounted for by plotting the effects on Ca^2+^ dissociation *versus* the amount of MyBP-C fragment bound, we continue to see dramatic differences (Fig. 3). At the concentrations we used (up to 2.3 μΜ for C0-C2 and C1-C2, and 10 μM for C0-C1), all fragments achieved similar binding (∼1–2 cMyBP-C per tropomyosin). Thus, differences in thin filament occupancy alone do not account for the fragment-dependent differences in Ca^2+^ dissociation kinetics. For a cartoon depiction and summary of construct and phosphorylation effects on *k*_off_ IC_50_, *k*_off_, and *k*_on_ values, see Figure 4 (additional cMyBP-C ratios and *p*-values are summarized in Tables S1 and S2 of the Supplemental Data).

**Figure 3:**
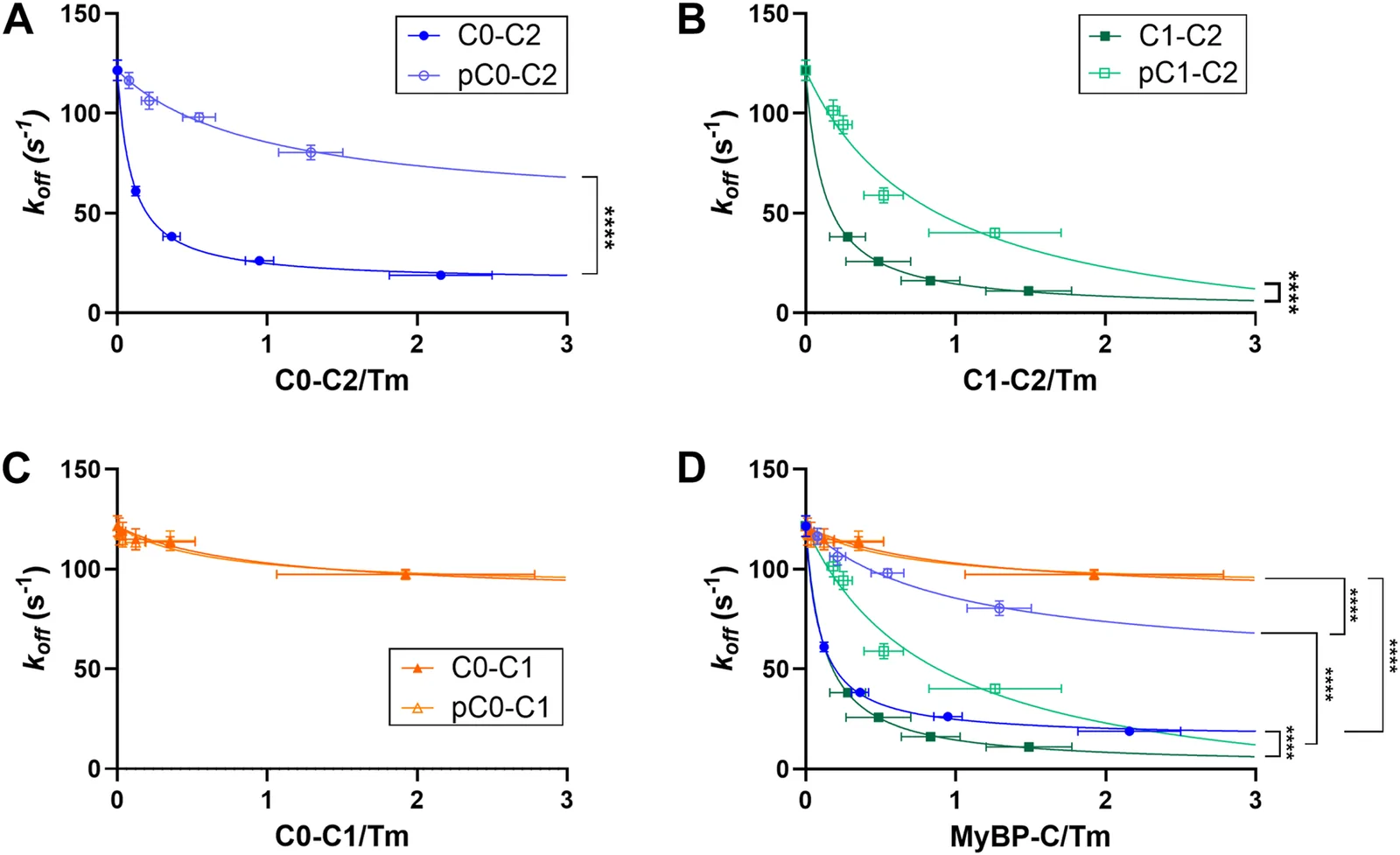
**Ca^2+^ dissociation as a function of bound cMyBP-C N-terminal fragment.** Dependence of Ca^2+^ dissociation rates (*k*_*off*_) on the amount of bound unphosphorylated (*filled symbols*) or phosphorylated (*open symbols*) (*A*) C0-C2, (*B*) C1-C2 and, (*C*) C0-C1. *D*, superimposed comparison of all fragments. Data are presented as mean ± S.D. (N = 2 protein preparations, n = 5–6 for cosedimentation assays and 9–13 for stopped-flow Ca^2+^ dissociation). ∗∗∗∗*p* ≤ 0.0001 comparing least squares fits using extra sum-of-squares F test. cMyBP-C, cardiac myosin-binding protein C.

**Figure 4:**
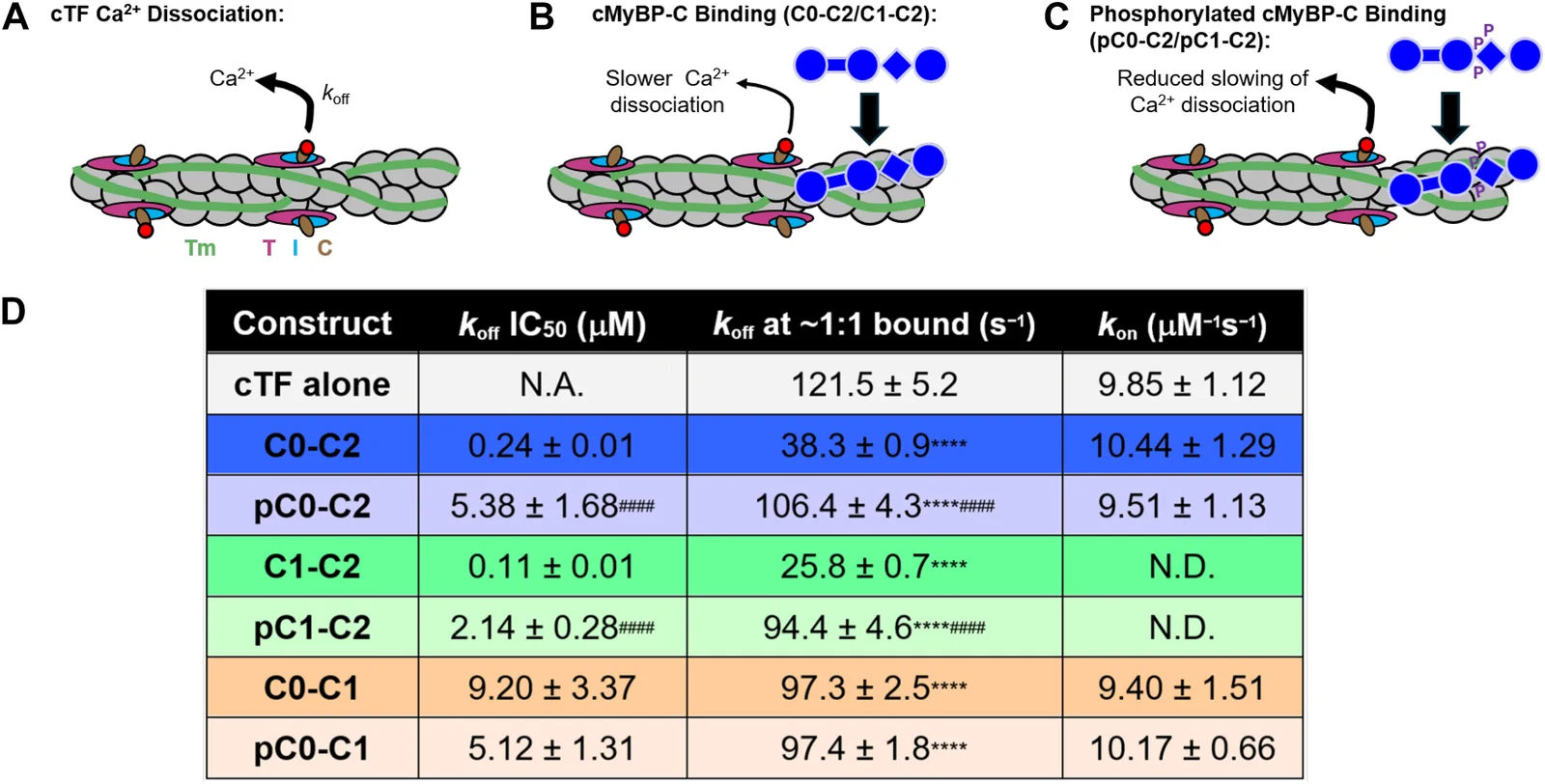
**Model and summary of C0-C2/C1-C2 phosphorylation-sensitive effects on Ca^2+^ dissociation in cTF.***A*–*C*, schematic models summarizing the proposed mechanism in which C0-C2 and C1-C2 slow Ca^2+^ dissociation *via* interactions with actin/Tm, whereas phosphorylation of the M-domain reduces this effect. *D*, summary of cMyBP-C fragment and phosphorylation effects on *k*_off_ IC_50_, *k*_off_, and *k*_on_. NA, not applicable; ND, not determined.∗∗∗∗*p* < 0.0001 *versus* cTF alone; ####*p* < 0.0001 *versus* the corresponding unphosphorylated construct. cTF, cardiac thin filament; cMyBP-C, cardiac myosin-binding protein C.

Summary data corrected for binding based on cosedimentation assays (Fig. S2) are shown in Figure 3. IC_50_ values (μM) derived separately from a least-squares fits of *k*_off_ *versus* fragment concentration differed significantly among fragments (*p* < 1.0 × 10^−15^) and phosphorylation states (*p* < 1.0 × 10^−15^): C0-C2 (-PKA, 0.24 ± 0.01; +PKA, 5.4 ± 1.7), C1-C2 (-PKA, 0.11 ± 0.01; +PKA, 2.1 ± 0.3). The modest effect of C0-C1, despite achieving comparable thin filament occupancy at higher concentrations, indicates that inclusion of the M-domain/C2 region is associated with substantially greater slowing of Ca^2+^ dissociation (Fig. 3; see also Fig. S3 for the modest C0-C1 effects at lower fragment:Tm ratios).

### cMyBP-C N-terminal effects are not observed with isolated troponin

To determine whether the slowing of Ca^2+^ dissociation requires the assembled actin/Tm-containing thin filament, we performed analogous stopped-flow experiments using isolated troponin complexes. In contrast to cTFs, cMyBP-C fragments had no effect on Ca^2+^ dissociation from cTn alone (47.2 ± 0.4 s^−1^, *p* = 0.99 for 1:1 C0-C2) (Fig. 5*A*), indicating that modulation requires the assembled actin/Tm-containing thin filament context.

**Figure 5:**
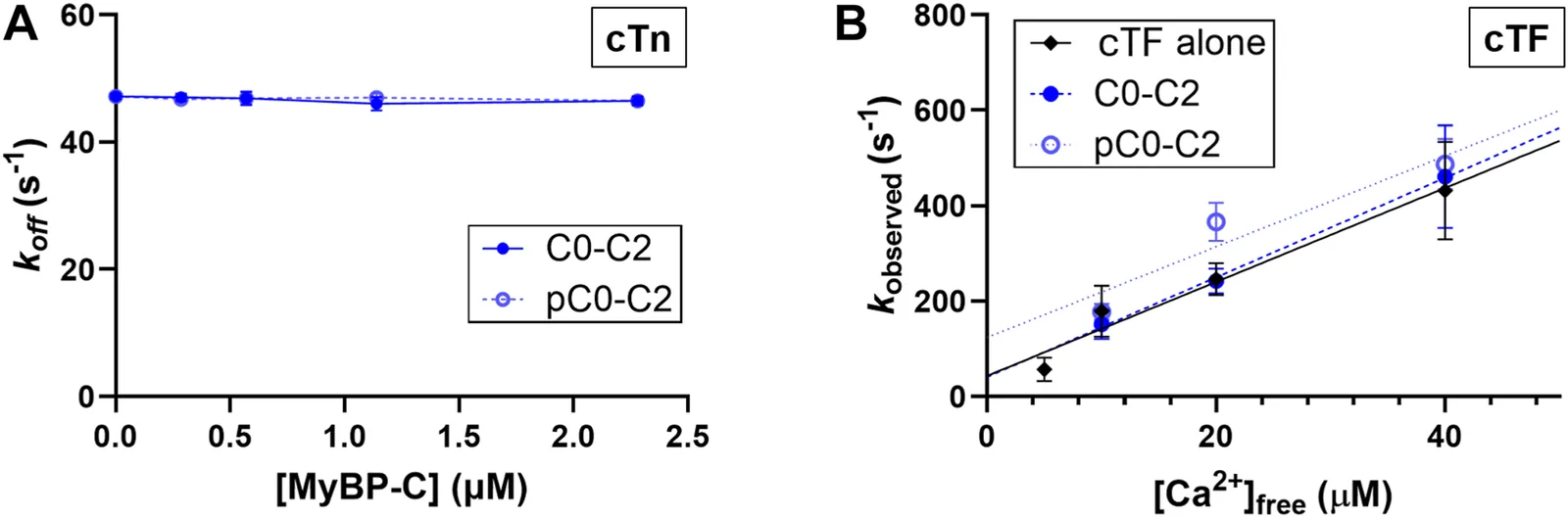
**N-terminal cMyBP-C control experiments of Ca^2+^ dissociation kinetics in isolated troponin and Ca^2+^ association kinetics in cardiac thin filaments (cTFs).***A*, unphosphorylated and phosphorylated C0-C2 do not alter Ca^2+^ dissociation in isolated cTn. *B*, Ca^2+^ association kinetics (*k*_*obs*_) in cTFs; data were fitted with a linear regression model to derive *k*_*on*_. Data are presented as mean ± SD (N = 2 protein preparations, n = 4–8). Statistical significance was determined by (*A*) two-way ANOVA followed by Tukey’s *post hoc* test for multiple comparisons, and (*B*) statistical significance between curve fits was determined by an extra sum-of-squares F test. cMyBP-C, cardiac myosin-binding protein C.

### cMyBP-C N-terminal domains do not affect Ca^2+^ association kinetics

We next examined whether cMyBP-C influences Ca^2+^ association with cTnC. Samples containing 5 μM EGTA were rapidly mixed with increasing free Ca^2+^ concentrations (0–40 μM), and IAANS-cTnC fluorescence changes were monitored. Observed first-order association rates (*k*_obs_) were plotted against free [Ca^2+^], and the slope was used to determine the second-order association rate constant (*k*_on_). The cMyBP-C fragments tested for association kinetics did not alter Ca^2+^-dependent association kinetics (Fig. 5*B*). Intrinsic *k*_*on*_ values were indistinguishable (*p* = 0.87) from cTF alone (9.9 ± 1.1 μM^−1^s^−1^) for both unphosphorylated (C0-C2, 10.4 ± 1.3 μM^−1^s^−1^) and phosphorylated (pC0-C2, 9.5 ± 1.1 μM^−1^s^−1^) fragments, indicating selective modulation of Ca^2+^ dissociation.

## Discussion

### Mechanistic insights into how cMyBP-C N-terminal domains affect Ca^2+^ dissociation from cTFs

Prior work has established that cMyBP-C N-terminal fragments bind actin-containing thin filaments and can alter tropomyosin position on actin, consistent with modulation of thin filament regulatory state (3). Structural and biochemical studies have shown that cMyBP-C N-terminal domains interact with actin and/or tropomyosin, while motility and muscle studies indicate that cMyBP-C can modulate thin filament sliding, Ca^2+^ sensitivity, force development, and relaxation (3, 4, 11, 12, 13, 14, 15). Phosphorylation provides an additional regulatory layer by weakening or altering N-terminal interactions with actin, myosin, and/or thin filaments (16, 17, 18, 19) in part due to changes in conformational dynamics (16, 17). Our results extend this work by demonstrating that N-terminal cMyBP-C fragments can also modulate the kinetics of Ca^2+^ dissociation from thin filament-bound cTnC.

Both C0-C2 and C1-C2 slowed Ca^2+^ dissociation in a concentration-dependent manner, whereas C0-C1 produced only a modest effect (Figure 1, Figure 2, Figure 3, S3, Tables S1, and S2). Although C0-C1 bound weakly at the fragment:Tm ratios used for C0-C2 and C1-C2 (Fig. S2), increasing the C0-C1 concentration to achieve comparable thin filament occupancy produced only a modest slowing of Ca^2+^ dissociation (Fig. 3, *C* and *D* and 4*D*). Thus, differences in kinetic effects among the N-terminal fragments cannot be explained solely by differences in thin filament binding. The substantially greater effects of C0-C2 and C1-C2 compared with C0-C1 suggest an important contribution from the interactions involving the M-domain/C2 region to modulation of cTnC Ca^2+^ dissociation, although these experiments do not isolate the contribution of an individual domain. PKA-mediated phosphorylation markedly attenuated the effects of C0-C2 and C1-C2, further indicating that both N-terminal domain composition and phosphorylation state influence the magnitude of this regulation (Figure 2, Figure 3, Figure 4). Thus, cMyBP-C N-terminal domains can modulate a kinetic step associated with cTF deactivation in a domain- and phosphorylation-sensitive manner.

These functional differences cannot be explained simply by variations in thin filament binding. Cosedimentation assays, at the fragment concentrations we used, demonstrated broadly similar maximal binding of all fragments (∼1–2 cMyBP-C molecules per Tm) (Fig. S2). Moreover, replotting kinetic effects as a function of bound protein showed that constructs containing the M- and C2 domains retained the strongest effects. These findings indicate that differences in Ca^2+^ dissociation kinetics reflect domain-dependent effects of bound cMyBP-C rather than differences in thin filament occupancy (Fig. 3).

Domain-specific comparisons further localize the strongest functional effect to constructs containing the C1-M-C2 region. C1-C2 produced a greater effect than C0-C2, suggesting that inclusion of C0 and the proline/alanine-rich linker may partially attenuate this response, whereas removal of the M and C2 domains in C0-C1 markedly reduced the effect (Figure 1, Figure 2, Figure 3, Figure 4). Consistent with recent structural studies showing that the C1 domain can stabilize the cTn core in an activated conformation (5), our results support a model in which binding of the cMyBP-C N-terminal region stabilizes a thin filament regulatory configuration that favors retention of Ca^2+^ at cTnC and slows its dissociation.

The reduced slowing observed following PKA treatment is consistent with phosphorylation-sensitive regulation of cMyBP-C N-terminal thin filament interactions (18, 20). Although performed under myosin-free conditions, this phosphorylation-sensitive modulation of thin filament Ca^2+^ dissociation parallels established effects of cMyBP-C phosphorylation on cross-bridge cycling kinetics and myocardial relaxation (21, 22). Similar to phosphorylated cTnI (or phosphomimetic cTnI-S23D/S24D), which accelerates cTF deactivation (10, 23, 24, 25), phosphorylation of cMyBP-C may provide an additional mechanism for modulating thin filament deactivation kinetics.

Trace amounts of the PKA catalytic subunit could not be entirely excluded following extensive dialysis and buffer exchange; however, ATP was removed before addition of enzymatically phosphorylated cMyBP-C fragments to cTFs, and therefore residual PKA would not be expected to phosphorylate cTnI. cTnI phosphorylation was not directly assessed in the final reaction mixtures.

The absence of an effect on isolated troponin indicates that cMyBP-C does not measurably alter cTnC Ca^2+^ dissociation through troponin alone under these conditions. Instead, the observed effect requires additional components of the assembled thin filament. Prior studies demonstrate that cMyBP-C N-terminal domains bind actin and tropomyosin and can alter tropomyosin position and actin structural dynamics (3). Thus, the present results are consistent with an indirect allosteric mechanism involving actin and/or tropomyosin. In the absence of strongly bound myosin, such an effect would be most consistent with modulation of the blocked-to closed-state regulatory equilibrium rather than stabilization of the fully activated myosin-bound state. However, the present experiments do not distinguish tropomyosin-dependent from tropomyosin-independent pathways, and direct attribution of the kinetic effect to tropomyosin would require additional experiments.

### Study limitations and future directions

#### Myosin

A key limitation is that Ca^2+^ dissociation was measured in myosin-free reconstituted cTFs. These experiments therefore define cMyBP-C-dependent effects in the absence of strong actomyosin interactions and are most directly relevant to regulation in the blocked/closed-state thin filament equilibrium. They do not directly assess the myosin bound-state stabilized by myosin. Because strong myosin binding alters thin filament regulatory state and cTnC Ca^2+^ exchange, future experiments incorporating NEM-S1, rigor S1, or more complete myofilament systems are needed to determine whether myosin masks, amplifies, or otherwise modifies the effects observed here.

#### Mechanism and Tm

The molecular pathway coupling cMyBP-C binding to altered cTnC Ca^2+^ dissociation also remains unresolved. The lack of an effect on isolated troponin indicates that components of the intact actin/Tm-containing thin filament are required for the observed response, but does not distinguish between tropomyosin-dependent and tropomyosin-independent mechanisms. Experiments using alternative or engineered tropomyosins with altered actin-binding or mechanical properties could directly test the contribution of tropomyosin.

#### Skeletal muscle comparison

Comparison with skeletal thin filament systems will also be informative because skeletal TnC contains two functional regulatory Ca^2+^-binding sites, whereas cardiac TnC contains a single functional regulatory site, and skeletal MyBP-C isoforms differ from cMyBP-C in domain composition and regulation. Such studies could determine whether the kinetic effects observed here represent a cardiac-specific mechanism or a more general property of MyBP-C/thin filament regulation.

In summary, these findings identify a phosphorylation-sensitive effect of cMyBP-C N-terminal domains on Ca^2+^ dissociation from cTnC in myosin-free reconstituted cTFs. The absence of an effect on isolated troponin indicates that additional thin filament components are required and supports a mechanism involving allosteric coupling through actin and/or tropomyosin. In the myosin-free system studied here, this effect is consistent with modulation of a kinetic step associated with thin filament deactivation and the blocked/closed-state regulatory equilibrium.

## Experimental procedures

All reagents were purchased from Thermo Fisher Scientific unless noted otherwise.

### *In vitro* cTF reconstitution

Expression and purification of cardiac troponin (cTn) subunits were performed as recently described (10, 26). Human cTnC contains two native cysteines, Cys35 and Cys84; both were substituted with serine residues (C35S and C84S), and T53 C was introduced to enable site-specific labeling with a fluorescent dye. The IAANS-labeled cTnC^T53C^ reporter strategy, developed, and extensively applied by Davis *et al*., monitors Ca^2+^ binding and exchange in troponin complexes and reconstituted thin filaments with minimal perturbation of cTnC function and reports Ca^2+^-dependent conformational changes in the cTnC regulatory domain (9, 27, 28, 29). cTnC^T53C^ was labeled with a fivefold molar excess of the thiol-reactive, environmentally-sensitive dye IAANS (Toronto Research Chemicals) as described previously (27), with slight modifications (30). Methods for purification of rabbit F-actin (31) and human tropomyosin (Tm) (20) have also been described.

cTFs were reconstituted at a molar ratio of 7:1:0.88 (F-actin:Tm:cTn) in Buffer A containing 10 mM 3-(N-morpholino)propanesulfonic acid (MOPS), 150 mM KCl, 3 mM MgCl_2_, and 1 mM DTT, pH 7.0. F-Actin and Tm were mixed and incubated on ice for 40 min, then cTn-IAANS was added and the mixture incubated on ice for an additional 20 min. Buffer A was then added to dilute the cTFs to a final working concentration of 0.5 μM IAANS.

### Preparation of recombinant human cMyBP-C fragments and PKA treatment

Human cMyBP-C fragments C0-C2 and C0-C1 were expressed, purified, and phosphorylated by PKA treatment as described (31). Both cMyBP-C fragments were further purified by size exclusion chromatography as recently described (32). Following PKA treatment, cMyBP-C fragments were dialyzed/buffer exchanged into stopped-flow experimental buffer to remove all ATP before addition to reconstituted cTFs.

To generate the C1-C2 fragment, pET45b vectors encoding *E. coli-*optimized codons for the C0-C2 region of human cMyBP-C with an N-terminal His tag and a tobacco etch virus protease (TEVp) cleavage site (GenScript) were truncated using primers to remove the C0 domain and the Pro/Ala-rich linker, while retaining the TEVp-cleavable His tag. Following TEVp cleavage, the resulting C1-C2 construct began with the sequence GDPIGLFVMR. Because C1-C2 was highly pure after purification on His60 Ni Superflow resin, additional purification by size exclusion chromatography was not required. We PKA-treated C1-C2 using the same phosphorylation protocol as for C0-C2 and C0-C1.

### Stopped-flow Ca^2+^ dissociation kinetics

Ca^2+^ dissociation rates (*k*_*off*_) were measured using an Applied Photophysics SX20 stopped-flow spectrometer at 15 °C with a dead time of 1.25 ms. Prior to stopped-flow measurements, cTFs were mixed with 0 to 10 μM cMyBP-C fragments and incubated for 10 min on ice.

For dissociation measurements, samples containing 200 μM CaCl_2_ were rapidly mixed with 12 mM EGTA. A premixing concentration of 0.3 μM IAANS-labeled cTnC (cTn-IAANS) was used for cTn-IAANS alone, and 0.5 μM IAANS (premixing) was used for cTF preparations. Excitation was set to 330 nm using a monochromator, and fluorescence emission was monitored at 450 nm for cTn-IAANS alone or at 510 nm for cTFs.

Data traces, each representing an average of 3 to 5 serial injections, were fitted with a single-exponential decay function to determine the observed *k*_*off*_ values. Reported values represent the mean ± SD.

### Stopped-flow Ca^2+^ association kinetics

Ca^2+^ association rates (*k*_*on*_) of cTF-IAANS were measured in the same stopped-flow spectrometer and excitation/emission settings described previously. To remove contaminating Ca^2+^, 5 μM EGTA was added to each cTF sample. Prior to stopped-flow experiments, cTFs were mixed with 0.57 μM cMyBP-C (1:1 cMyBP-C:Tm ratio) fragments and incubated for 10 min on ice.

For association measurements, Ca^2+^-free samples were rapidly mixed with buffer containing increasing concentrations of Ca^2+^ (2.5–40 μM). As with dissociation experiments, each data trace represented the average of 3 to 5 sequential injections and was fitted with a single-exponential function to determine the observed first-order association rate (*k*_*obs*_) at each Ca^2+^ concentration. Reported values represent the mean *k*_*obs*_ at each Ca^2+^ concentration.

Observed *k*_*obs*_ values were plotted as a function of Ca^2+^ concentration, and the resulting data were fitted by linear regression. The mean slope (±SD) of each regression was reported as the rate constant for Ca^2+^ association (*k*_*on*_).

### cTF cosedimentation assays

cTF binding by cMyBP-C fragments was determined by cosedimentation and analyzed as described in (33), except that assays were performed in Buffer A.

### Statistics

Statistical analyses were performed in GraphPad Prism as specified in the individual figure legends. One- or two-way ANOVA with the indicated *post hoc* multiple-comparison test was used as appropriate for the experimental design, and curve fits were compared using an extra sum-of-squares F test. Statistical significance was defined as *p* < 0.05. Data are reported as mean ± SD from *N* ≥ 2 independent protein preparations (biological replicates). The definition of *n* (technical replicates; *e.g.*, the number of serial injection averages) is specified in the individual figure legends.

## Data availability

All data discussed are presented within the article.

## Supporting information

This article contains supporting information.

## Declaration of generative AI technologies in the writing process

The authors did not use generative AI technologies in the development of this article.

## Conflict of interest

The authors declare that they have no conflicts of interest with the contents of this article.

## Supporting information

Supplemental Data Figure S1 (Data traces of C0-C2 and C1-C2 effects on Ca^2+^ dissociation kinetics), Figure S2 (Cosedimentation binding), Figure S3 (C0-C1 on *k*_off_), Table S1 (Summary of kinetic parameters of varying cMyBP-C ratios), and Table S2 (Summary of kinetic statistics).
