# Supplementary material for: Myosin-binding protein C N-terminal domains slow Ca2+ dissociation from cardiac thin filaments
Source: J Biol Chem. 2026 Sep 23;302(11):113601. doi: 10.1016/j.jbc.2026.113601 (PMC13696821; doi:10.1016/j.jbc.2026.113601)
Supplement: Supplementary file 1 [file mmc1.pdf]

Supplemental Data

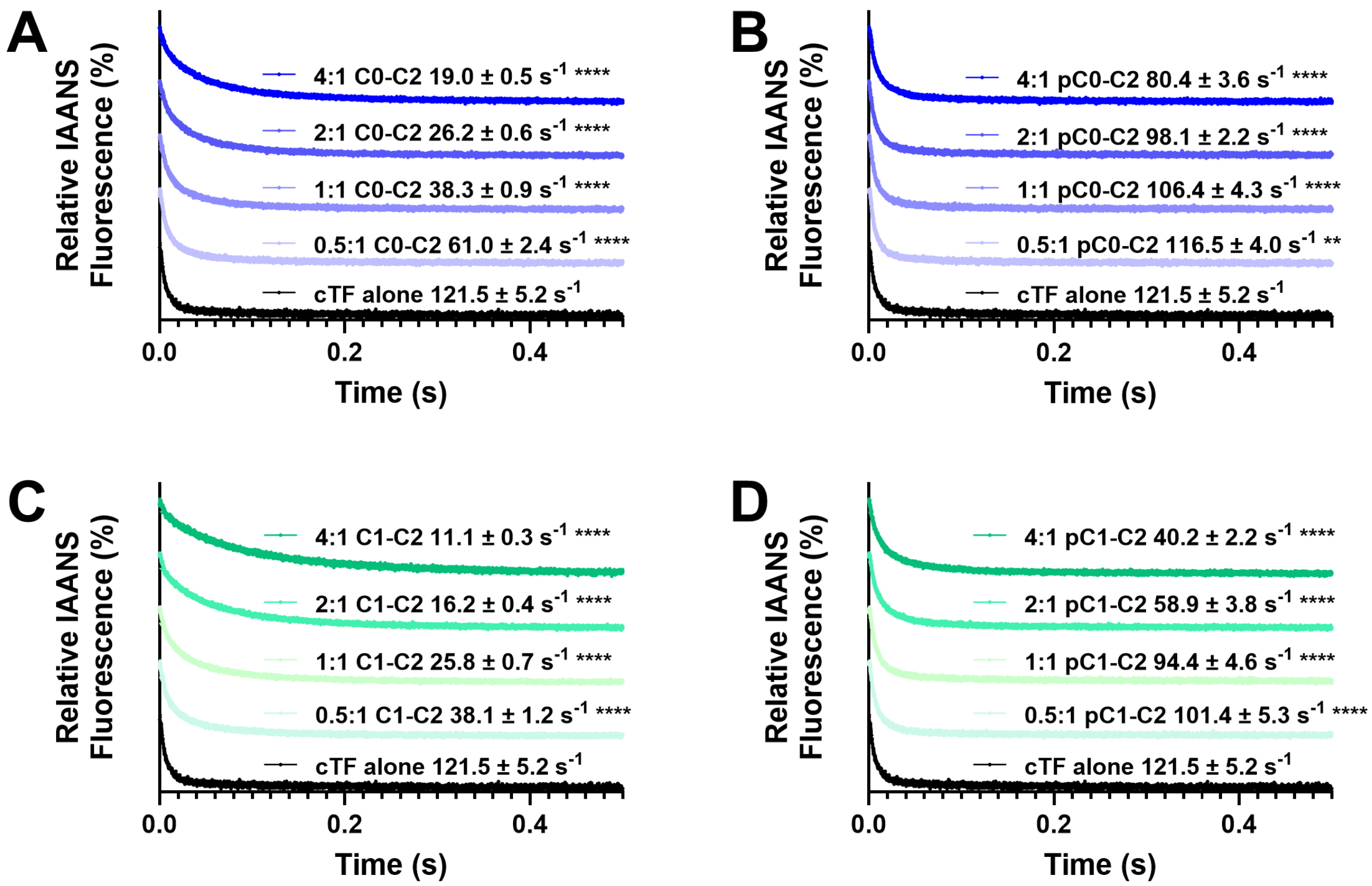

Supplemental Figure S1: Effects of C0-C2, C1-C2, and their phosphorylation on Ca^{2+} dissociation data traces. Vertically staggered representative IAANS fluorescence decays showing Ca^{2+} dissociation from cTnC in reconstituted cTFs in the presence of **(A)** unphosphorylated C0-C2 or **(B)** PKA-phosphorylated C0-C2 (pC0-C2) (blue/purple traces). Representative fluorescence decays for **(C)** unphosphorylated and **(D)** phosphorylated C1-C2 (green/teal traces). Ratios shown are C0-C2:Tm and C1-C2:Tm. Statistical significance was determined by two-way ANOVA followed by Tukey's post-hoc test for multiple comparisons: **$p \leq 0.01$ and ****$p \leq 0.0001$ for C0-C2/C1-C2 *vs.* cTF alone.

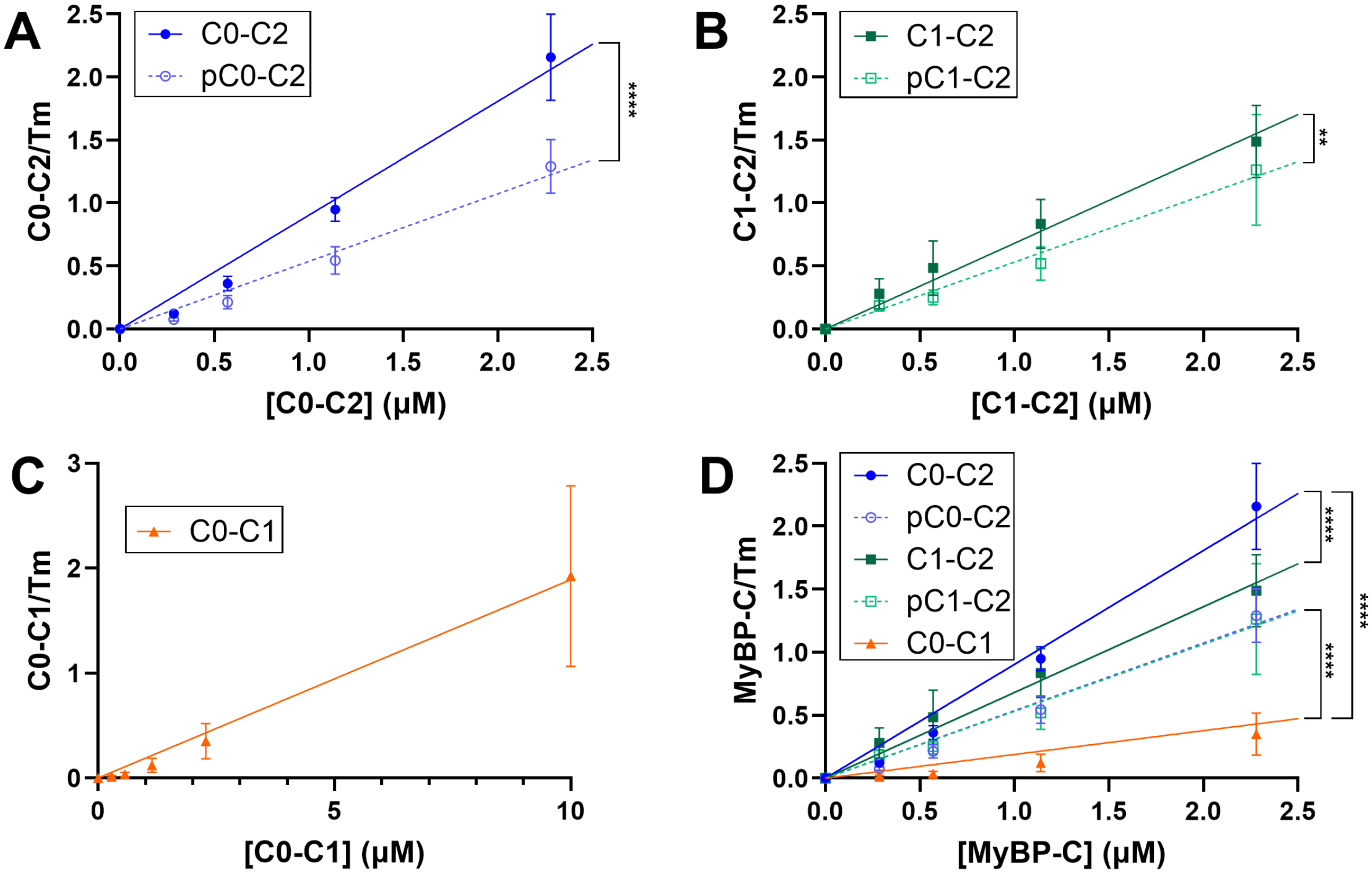

Supplemental Figure S2: Binding of cMyBP-C N-terminal fragments to cardiac thin filaments. Cosedimentation assays showing binding of unphosphorylated and phosphorylated **(A)** C0-C2 and **(B)** C1-C2, and **(C)** C0-C1 to cTFs, expressed as the molar ratio of cMyBP-C to tropomyosin (cMyBP-C/Tm). **(D)** Superimposed summary of fragments binding to cTFs. In D, the lines for phosphorylated C0-C2 and C1-C2 overlap. Data are presented as mean ± S.D. (N=2 protein preparations, n=5-6 for cosedimentation assays). $^{**}p \leq 0.01$ and $^{****}p \leq 0.0001$ for comparison of cMyBP-C phosphorylation or fragments. Statistical significance between curve fits was determined by an extra sum-of-squares F test.

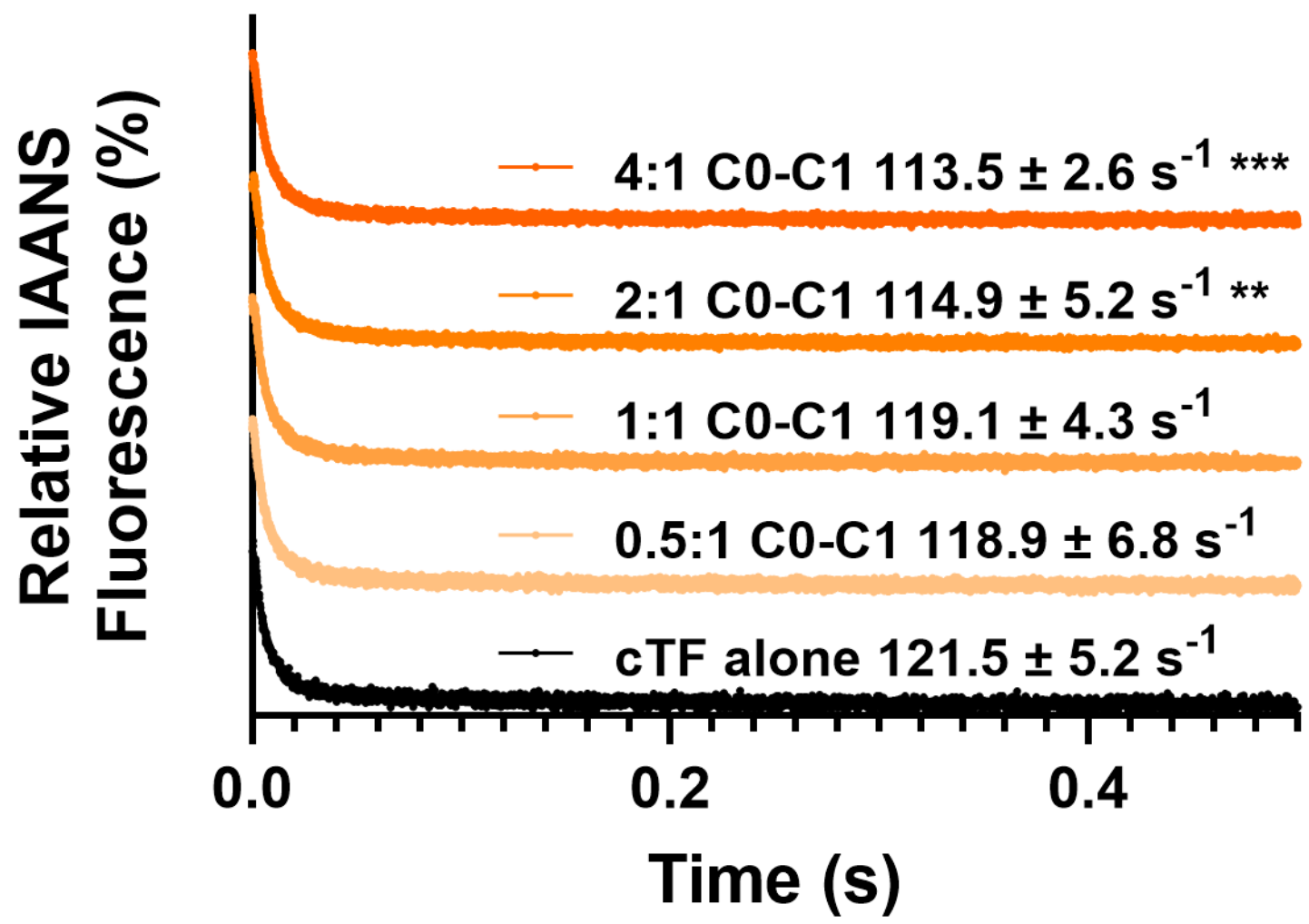

Supplemental Figure S3. C0-C1 produces only modest slowing of Ca^{2+} dissociation from cTnC in cTFs. Representative fluorescence decays for C0-C1. Data are presented as mean ± S.D. (N=2 protein preparations, n=9-13 injection averages). Ratios shown are C0-C1:Tm. Statistical significance was determined by two-way ANOVA followed by Tukey's post-hoc test for multiple comparisons: **p≤0.01 and ***p≤0.001 for C0-C1 *vs.* cTF alone.

Supplemental Table S1. Summary of kinetic parameters

Construct	k_{off} IC_{50} (μM)	Ratio to Tm	k_{off} (s^{-1})	k_{on} ($\mu M^{-1}s^{-1}$)
**cTF alone**	N.A.	N.A.	121.5 ± 5.2	9.85 ± 1.12
**C0-C2**	0.24 ± 0.01	0.5:1	61.0 ± 2.4****	N.D.
		1:1	38.3 ± 0.9****	10.44 ± 1.29
		2:1	26.2 ± 0.6****	N.D.
		4:1	19.0 ± 0.5****	N.D.
**pC0-C2**	5.38 ± 1.68####	0.5:1	116.5 ± 4.0**####	N.D.
		1:1	106.4 ± 4.3****####	9.51 ± 1.13
		2:1	98.1 ± 2.2****####	N.D.
		4:1	80.4 ± 3.6****####	N.D.
**C1-C2**	0.11 ± 0.01	0.5:1	38.1 ± 1.2****	N.D.
		1:1	25.8 ± 0.7****	N.D.
		2:1	16.2 ± 0.4****	N.D.
		4:1	11.1 ± 0.3****	N.D.
**pC1-C2**	2.14 ± 0.28####	0.5:1	101.4 ± 5.3****####	N.D.
		1:1	94.4 ± 4.6****####	N.D.
		2:1	58.9 ± 3.8****####	N.D.
		4:1	40.2 ± 2.2****####	N.D.
**C0-C1**	9.20 ± 3.37	0.5:1	118.9 ± 6.8	N.D.
		1:1	119.1 ± 4.3	9.40 ± 1.51
		2:1	114.9 ± 5.2**	N.D.
		4:1	113.5 ± 2.6***	N.D.
**pC0-C1**	5.12 ± 1.31	0.5:1	117.9 ± 3.2	N.D.
		1:1	114.5 ± 3.5**	10.17 ± 0.66
		2:1	113.3 ± 3.6***	N.D.
		4:1	114.2 ± 4.8**	N.D.

**p<0.01, ***p<0.001, ****p<0.0001 vs. cTF alone; $^{\#\#\#\#}$p<0.0001 vs. the corresponding unphosphorylated construct at the same Tm ratio. N.A., not applicable; N.D., not determined. See Table S2 for corresponding p-values.

Supplemental Table S2. Statistical comparisons for kinetic parameters

A. k_{off} values

Construct	Ratio to Tm	k_{off} (s^{-1})	p-value *vs.* cTF alone	p-value *vs.* unphosphorylated construct at the same Tm ratio	p-value *vs.* corresponding C0-C2 construct at the same Tm ratio and PKA condition
**cTF alone**	N.A.	121.5 ± 5.2	N.A.	N.A.	N.A.
**C0-C2**	0.5:1	61.0 ± 2.4	5.2×10^{-14}	N.A.	N.A.
	1:1	38.3 ± 0.9	5.2×10^{-14}	N.A.	N.A.
	2:1	26.2 ± 0.6	5.2×10^{-14}	N.A.	N.A.
	4:1	19.0 ± 0.5	5.2×10^{-14}	N.A.	N.A.
**pC0-C2**	0.5:1	116.5 ± 4.0	7.9×10^{-3}	5.2×10^{-14}	N.A.
	1:1	106.4 ± 4.3	8.6×10^{-14}	5.2×10^{-14}	N.A.
	2:1	98.1 ± 2.2	5.2×10^{-14}	5.2×10^{-14}	N.A.
	4:1	80.4 ± 3.6	5.2×10^{-14}	5.2×10^{-14}	N.A.
**C1-C2**	0.5:1	38.1 ± 1.2	$<1 \times 10^{-15}$	N.A.	$<1 \times 10^{-15}$
	1:1	25.8 ± 0.7	$<1 \times 10^{-15}$	N.A.	9×10^{-15}
	2:1	16.2 ± 0.4	$<1 \times 10^{-15}$	N.A.	8.6×10^{-14}
	4:1	11.1 ± 0.3	$<1 \times 10^{-15}$	N.A.	9.1×10^{-11}
**pC1-C2**	0.5:1	101.4 ± 5.3	$<1 \times 10^{-15}$	$<1 \times 10^{-14}$	2.1×10^{-13}
	1:1	94.4 ± 4.6	$<1 \times 10^{-15}$	$<1 \times 10^{-14}$	1.3×10^{-9}
	2:1	58.9 ± 3.8	$<1 \times 10^{-15}$	$<1 \times 10^{-14}$	5.2×10^{-14}
	4:1	40.2 ± 2.2	$<1 \times 10^{-15}$	$<1 \times 10^{-14}$	5.2×10^{-14}
**C0-C1**	0.5:1	118.9 ± 6.8	0.92	N.A.	5.2×10^{-14}
	1:1	119.1 ± 4.3	0.96	N.A.	5.2×10^{-14}
	2:1	114.9 ± 5.2	7.7×10^{-3}	N.A.	5.2×10^{-14}
	4:1	113.5 ± 2.6	3.4×10^{-4}	N.A.	5.2×10^{-14}
**pC0-C1**	0.5:1	117.9 ± 3.2	0.60	1	1
	1:1	114.5 ± 3.5	3.2×10^{-3}	0.23	5.5×10^{-5}
	2:1	113.3 ± 3.6	2.1×10^{-4}	1	1.5×10^{-13}
	4:1	114.2 ± 4.8	1.5×10^{-3}	1	5.2×10^{-14}

B. k_{off} IC_{50} values

Construct	IC_{50} (μM)	p-value *vs.* unphosphorylated construct	p-value *vs.* corresponding C0-C2 construct at the same PKA condition
**C0-C2**	0.24 ± 0.01	N.A.	N.A.

**pC0-C2**	5.38 ± 1.68	<1 x 10^{-15}	N.A.
**C1-C2**	0.11 ± 0.01	N.A.	<1 x 10^{-15}
**pC1-C2**	2.14 ± 0.28	<1 x 10^{-15}	<1 x 10^{-15}
**C0-C1**	9.20 ± 3.37	N.A.	<1 x 10^{-15}
**pC0-C1**	5.12 ± 1.31	0.42	<1 x 10^{-15}

C. k_{on} values

**Construct**	**k_{on} (μ$M^{-1}s^{-1}$)**	**p-value *vs.* cTF alone**	**p-value *vs.* unphosphorylated construct**	**p-value *vs.* corresponding C0-C2 construct at the same PKA condition**
**cTF alone**	9.85 ± 1.12	N.A.	N.A.	N.A.
**C0-C2**	10.44 ± 1.29	0.73	N.A.	N.A.
**pC0-C2**	9.51 ± 1.13	0.84	0.59	N.A.
**C1-C2**	N.D.	N.D.	N.D.	N.D.
**pC1-C2**	N.D.	N.D.	N.D.	N.D.
**C0-C1**	9.40 ± 1.51	0.81	N.A.	0.61
**pC0-C1**	10.17 ± 0.66	0.80	0.62	0.60

N.A., not applicable; N.D., not determined.
